# Live Vaccinia Virus-Coated Microneedle Array Patches for Smallpox Vaccination and Stockpiling

**DOI:** 10.3390/pharmaceutics13020209

**Published:** 2021-02-03

**Authors:** In-Jeong Choi, Hye-Ran Cha, Su Jin Hwang, Seung-Ki Baek, Jae Myun Lee, Seong-O Choi

**Affiliations:** 1 QuadMedicine R&D Centre, QuadMedicine, Inc., Seongnam 13209, Korea; cij@quadmedicine.com (I.-J.C.); bsk@quadmedicine.com (S.-K.B.); 2 Department of Microbiology and Immunology, Institute for Immunology and Immunological Diseases, Graduate School of Medical Science, Brain Korea 21 Project, Yonsei University College of Medicine, Seoul 03722, Korea; hrcha@yuhs.ac (H.-R.C.); hapisujin@yuhs.ac (S.J.H.)

**Keywords:** smallpox, microneedle, vaccinia virus, coating, stability, immune response

## Abstract

Although smallpox has been eradicated globally, the potential use of the smallpox virus in bioterrorism indicates the importance of stockpiling smallpox vaccines. Considering the advantages of microneedle-based vaccination over conventional needle injections, in this study, we examined the feasibility of microneedle-based smallpox vaccination as an alternative approach for stockpiling smallpox vaccines. We prepared polylactic acid (PLA) microneedle array patches by micromolding and loaded a second-generation smallpox vaccine on the microneedle tips via dip coating. We evaluated the effect of excipients and drying conditions on vaccine stability in vitro and examined immune responses in female BALB/c mice by measuring neutralizing antibodies and interferon (IFN)-γ-secreting cells. Approximately 40% of the virus titer was reduced during the vaccine-coating process, with or without excipients. At −20 °C, the smallpox vaccine coated on the microneedles was stable up to 6 months. Compared to natural evaporation, vacuum drying was more efficient in improving the smallpox vaccine stability. Microneedle-based vaccination of the mice elicited neutralizing antibodies beginning 3 weeks after immunization; the levels were maintained for 12 weeks. It significantly increased IFN-γ-secreting cells 12 weeks after priming, indicating the induction of cellular immune responses. The smallpox-vaccine-coated microneedles could serve as an alternative delivery system for vaccination and stockpiling.

## 1. Introduction

Smallpox is caused by the variola virus, an enveloped double-stranded DNA virus belonging to the genus *Orthopoxvirus*. It is a highly contagious and morbid disease with approximately 30% mortality [1,2,3]. Following an organized vaccination program, the World Health Organization declared the global eradication of the disease in 1980. In South Korea, the smallpox vaccination program has been discontinued since 1979. However, there is an increased possibility that the smallpox virus could be used as a bioterrorism weapon, thus indicating the need for stockpiling smallpox vaccine [4,5]. Although first-generation smallpox vaccines saved millions of lives and achieved the eradication of smallpox, they presented limitations such as serious adverse events, unwanted immune responses to calf-derived materials, and bovine prion transmission [6]. To mitigate the side effects of the first-generation vaccines, a cell culture-derived smallpox vaccine (CJ-50300) was developed by a Korean pharmaceutical company and licensed by the Korea Food and Drug Administration in 2008 [7]. This vaccine is inoculated by puncturing the skin using a bifurcated needle [8,9]; however, vaccination using bifurcated needles has raised concerns regarding needle-related injuries and disease transmission, as well as biohazardous sharp waste generation.

Microneedles are microstructures that facilitate the delivery of molecules into the epidermal and/or dermal layer of the skin and can be used as a highly promising delivery system for vaccination [10,11,12,13,14]. There are different types of microneedles, including coated microneedles, dissolving microneedles, and hollow microneedles, depending on the morphology and delivery strategy. Typically, microneedles exhibit a conical or pyramidal shape with 1:1~1:3 aspect ratio (width to height), and they are formulated using biocompatible materials via various microfabrication techniques [10,15,16,17,18]. Although most vaccines have been administered through either intramuscular (IM) or subcutaneous (SC) routes using hypodermic needles [19,20], cutaneous immunization could serve as a superior effective approach for preventing pathogenic infection and facilitating dose sparing for multiple vaccines compared to IM or SC administration [21,22,23,24]. The skin is an attractive site for vaccination due to the abundance of antigen-presenting cells (APCs), including Langerhans cells, dermal dendritic cells, macrophages, and monocytes, as well as accessory cells such as keratinocytes [21,25]. These APCs recognize, uptake, and present foreign antigens to T- and B-cells in the draining lymph nodes to initiate adaptive immune responses. In addition to the advantage of cutaneous immunization, microneedles can resolve several issues associated with the use of hypodermic needles, such as pain and needlestick injuries, as well as the requirement for trained personnel, appropriate needle disposal, and expensive logistics. Moreover, solidified vaccines contained in microneedles are considerably more stable at increased temperatures than liquid-state vaccines, thereby reducing economic burden related to vaccine storage, transport, and distribution [26,27,28,29,30].

On the basis of the results from previous studies, we hypothesized that smallpox vaccination using microneedles would address the problems associated with the use of bifurcated needles and induce both humoral and cell-mediated immune responses [31,32]. In this study, we selected a coated microneedle system, among various microneedle platforms, for smallpox vaccination due to its higher mechanical strength, faster drug delivery, and easier fabrication process compared with other microneedle types [33,34]. We prepared second-generation smallpox-vaccine-coated microneedles by exploring various formulations, fabrication processes, and storage conditions and evaluated their efficacy in mice by measuring neutralizing antibodies and interferon (IFN)-γ secreting cells.

## 2. Materials and Methods

### 2.1. Vaccina Virus Vaccine

The second-generation vaccinia virus vaccine (CJ-50300) was kindly provided by the Korea National Institute of Health (KNIH). CJ-50300 was derived from the vaccinia virus strain ATCC VR-118 (originating from the New York City Board of Health vaccinia strain), which was adapted to replicate in MRC-5 cells under serum-free conditions without plaque purification [7].

### 2.2. Preparation of Coating Solution

In this study, pharmaceutical-grade polyvinyl alcohol (PVA; Gohsenol™ EG-30 PW; Nippon Gohsei Europe GmbH, Dusseldorf, Germany) and trehalose (Sigma-Aldrich Korea, Yongin, South Korea) were used as excipients. Four different coating solutions were prepared in phosphate-buffered saline (PBS; pH 7.4; Thermo Fisher Scientific, Waltham, MA, USA), as summarized in Table 1. The prepared solutions were stored at 4 °C for 2 h prior to use.

### 2.3. Fabrication of Coated Microneedles

A microneedle master, composed of 97 obelisk microneedles (800 μm height, 370 μm width, 400 μm gap between needles) in a circular base (1 cm diameter), was prepared by micromilling [35]. A microneedle mold was prepared by casting polydimethylsiloxane (PDMS, Sylgard^®^ 184; Dow Corning, Midland, MI, USA) over the master followed by curing at 70 °C for 1 h. Polymeric microneedles were subsequently replicated through casting polylactic acid (PLA; DURECT Corp., Birmingham, AL, USA) into the mold. PLA pellets were stacked on the mold and melted at 190 °C for 30 min in a vacuum oven. Bubbles generated during the PLA casting were removed by applying vacuum (approximately 70 kPa) several times. Once the molten PLA was filled in the mold cavity, the mold was cooled at 22 ± 2 °C. The PLA microneedles were subsequently detached from the mold.

**Figure 1:**
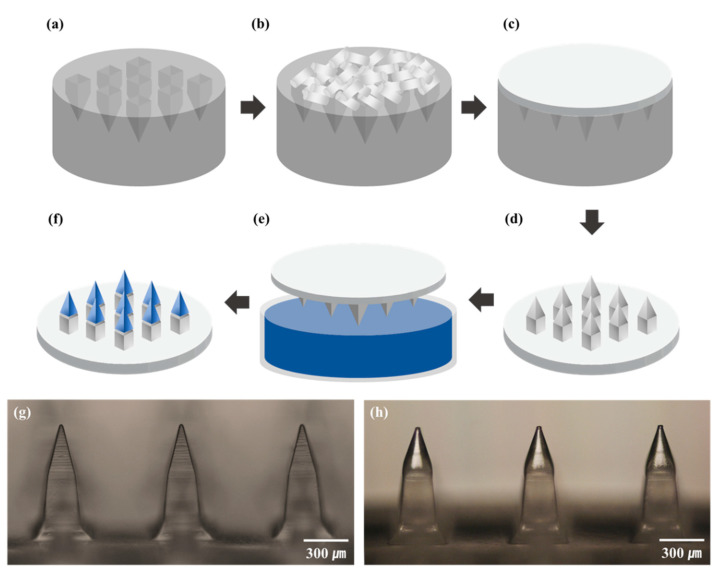
Fabrication process and results. (**a**) polydimethylsiloxane (PDMS) mold preparation, (**b**) polylactic acid (PLA) pellet loading, (**c**) PLA melt casting, (**d**) PLA microneedle demolding, (**e**) dip coating, (**f**) drying, (**g**) PLA microneedles, and (**h**) vaccine-coated microneedles.

A predetermined amount of vaccine was loaded on the microneedles through dip coating using a homemade apparatus. The coating solution, described in Table 1, was filled in a 400 μm-deep coating well, and the PLA microneedle attached to the linear actuator was dipped in the coating well at 1 mm/s for 1 s and pulled up at 1 mm/s. The coated microneedle was subsequently dried for 1 h either under ambient conditions (approximately 24 °C and 40% relative humidity) or under vacuum (5 × 10^−4^ Torr). Figure 1 shows the overall fabrication process and the fabricated microneedles.

To maintain the sterility of the smallpox vaccine-coated microneedles during fabrication as best as possible, all fabrication processes, including coating solution preparation, coating, and drying, were carried out in a biosafety cabinet, which was rigorously disinfected prior to microneedle fabrication. All materials, equipment, and consumables used for microneedle fabrication were also sterilized by a suitable technique, such as autoclave and filtration, considering the compatibility with the sterilization process.

### 2.4. In Vitro Vaccine Stability Test

The stability of vaccinia virus vaccine during drying and storage was examined in vitro. The plaque assay was used for measuring virus titers. To determine the vaccine stability upon drying, 10 μL of each formulation, described in Table 1, was spread on PLA surface having a diameter of 10 mm, which mimics the surface of the PLA microneedle, dried under ambient conditions, and reconstituted for the assay. We added 700 μL of PBS to F1 and F2, and 10 μL of each sample was used for the measurement (i.e., the estimated viral titer of each sample was 2.5 × 10^5^ PFU).

To examine the storage stability, vaccine-coated microneedles were prepared using each formulation, stored either at 4 °C or −20 °C for 1, 3, and 6 months, and were subjected to the assay.

For the plaque assay, the virus was recovered from the prepared samples through immersion of the samples in 1 mL of cell culture media (Opti-MEM™; Gibco, Carlsbad, CA, USA) at 4 °C for 2 h. Serial dilutions of the recovered virus solution were subsequently added to Vero cells in a 12-well plate. The final overlay was performed with 0.2% low-melting agarose (SeaPlaque™, Lonza, Basel, Switzerland) in Opti-MEM™, containing 2% fetal bovine serum (FBS; Gibco). The cells were cultured at 37 °C for 3 days, fixed, and stained with crystal violet mixture (0.13% crystal violet, 8% formaldehyde, 5% ethanol). Plaques were then counted under an optical microscope (ECLIPSE TS100; Nikon, Tokyo, Japan).

### 2.5. Animals and Vaccine Administration

Six-week-old female Balb/c mice were purchased from Orient Bio Inc. (Seongnam, South Korea) and maintained under specific pathogen-free conditions in the experimental facility at the International Vaccine Institute (IVI; Seoul, South Korea); they received sterilized food and water ad libitum. All animal experiments described were approved by the IVI Institutional Animal Care and Use Committees (approved code: PN 2020-012).

Prior to microneedle vaccination, the hair on the dorsal skin of the mice were removed using a depilatory cream (Nair™; Church and Dwight, Trenton, NJ, USA). Five mice per group were subsequently treated with either smallpox-vaccine-coated microneedles (mean titer: 3.3 × 10^5^ PFU) or blank microneedles. The microneedles were applied to the hair-free region using an adhesive patch and thumb pressure and were further affixed to the skin with a clamp for 30 min. Serum samples were collected from the retro-orbital plexus 1, 3, 6, 9, and 12 weeks after immunization.

### 2.6. Analysis of Neutralizing Antibody Responses

For analyzing neutralizing antibodies against the vaccinia virus, the plaque reduction neutralization test (PRNT) was performed. The PRNT50 titer was determined using the highest serum dilution inhibiting 50% or more of the plaques relative to the number of plaques in the absence of test serum. Serum samples were heat-inactivated at 56 °C for 30 min before use. Two-fold serial dilutions of the inactivated serum sample and virus samples containing 100 PFU were mixed and incubated at 37 °C for 2 h. Vero cells in 24-well plates (80–90% confluent, approximately 4 × 10^4^ cells/well) were treated with the incubated virus–serum mixtures. The final overlay was performed using 0.2% low-melting agarose in Opti-MEM™ media containing 2% FBS. The cells were cultured at 37 °C for 3 days, and the plaques were counted after fixing and staining the cells with crystal violet mixture (0.13% crystal violet, 8% formaldehyde, and 5% ethanol).

### 2.7. Analysis of Cell-Mediated Immune Responses

To examine cell-mediated immune responses, IFN-γ-secreting cells in splenocytes of the immunized mice were counted using the enzyme-linked immunospot (ELISPOT) assay (Mabtech, Inc., Cincinnati, OH, USA), according to manufacturer’s instructions. Single cell suspensions from the spleen tissue were obtained by mashing the spleen through the cell strainer using the plunger end of the syringe, and erythrocytes were lysed using Ammonium-Chloride-Potassium (ACK) buffer. For ELISPOT assay, 96-well plates (coated with capture antibody) were blocked with RPMI 1640 supplemented with 10% FBS; 5 × 10^5^ cells from each group were plated and stimulated with UV-inactivated vaccinia virus at 37 °C for 48 h in a 5% CO_2_ incubator. 3,3′,5,5′-tetramethylbenzidine (TMB) peroxidase substrate was used for color development, and IFN-γ-secreting cells were counted and visualized using an ELISPOT reader (ImmunoSpot^®^; Cellular Technology Limited, Cleveland, OH, USA). Data were analyzed by one-way analysis of variance (ANOVA) using GraphPad Prism software, version 8.0 (GraphPad Software Inc., San Diego, CA, USA).

### 2.8. Statistical Analysis

To determine statistical significance, a two-tailed Student’s *t*-test was performed when comparing two different conditions, and the one-way ANOVA was used when comparing multiple groups. A value of *p* < 0.05 was considered statistically significant.

## 3. Results and Discussion

### 3.1. Influence of Fabrication Process on Vaccine Stability

Commercial smallpox vaccine is a lyophilized live virus vaccine and needs to be maintained at −20 °C. To prepare smallpox-vaccine-coated microneedles, the vaccine is thawed, mixed with excipients, coated on the tip of microneedles, and solidified. During this fabrication process, the vaccine experiences phase transition twice (i.e., solid to liquid during thawing and liquid to solid during coating), which often causes vaccine instability. Therefore, it is necessary to examine the change in vaccine stability due to phase transition.

**Figure 2:**
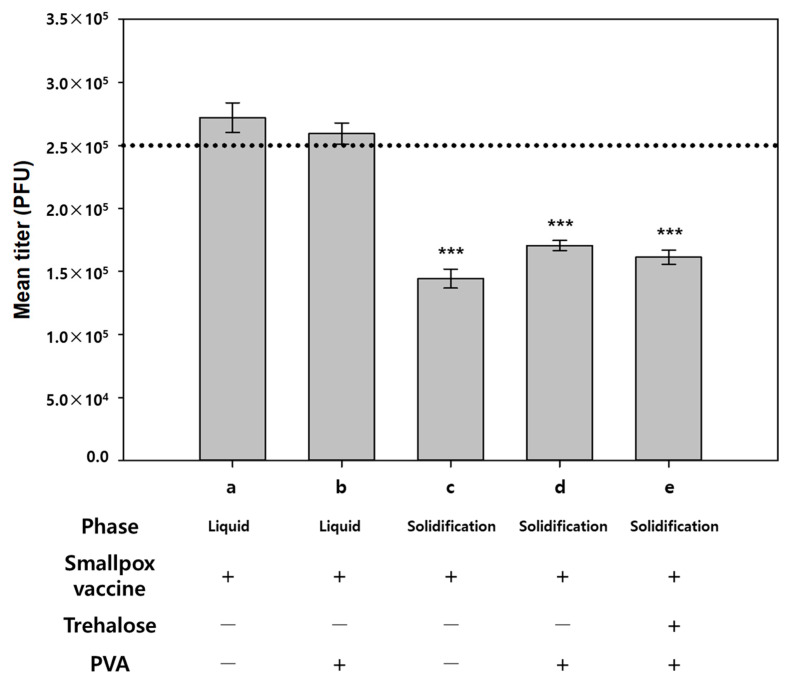
Virus titers of (**a**) smallpox vaccine (F1) (liquid), (**b**) smallpox vaccine + polyvinyl alcohol (F2) (PVA; liquid), (**c**) smallpox vaccine (F1) (reconstituted after solidification), (**d**) smallpox vaccine + PVA (F2) (reconstituted after solidification), and (**e**) smallpox vaccine + PVA + trehalose (F4) (reconstituted after solidification). Dotted line indicates the estimated virus titer. Statistical significance compared to liquid smallpox vaccine (**a**) was determined using Student’s *t*-test (*** *p* < 0.001).

We first examined the stability of the vaccine with or without PVA (F1 and F2 in Table 1) by measuring the viral titers. PVA was used as a viscosity enhancer, which is required for coating. As shown in Figure 2, there were no significant differences between the virus titers of F1 and F2 in the liquid state. However, solidification caused approximately 40% decrease in the virus titer for both cases. We next examined the effect of trehalose, a widely used vaccine stabilizer, on the stability of the smallpox vaccine during solidification. However, addition of trehalose produced no improvement in vaccine stability. This result suggests that the thawing and mixing processes minimally affect vaccine stability, whereas the drying process substantially reduced the viability of the vaccinia virus, regardless of the use of PVA and trehalose.

**Figure 3:**
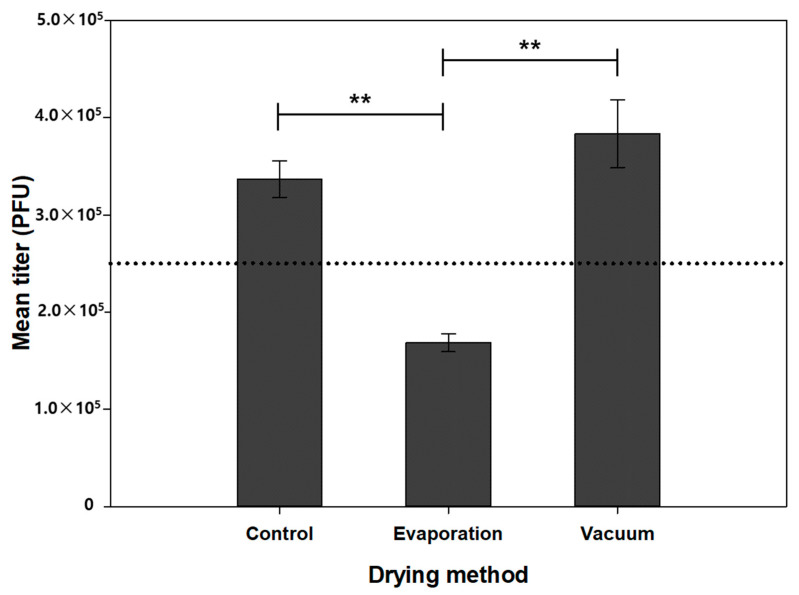
Effect of drying method on vaccine stability. Liquid formulation was used as a control. Statistical significance compared to the evaporation and vacuum drying samples was determined by a *t*-test (** *p* < 0.01).

As the stress induced during drying varies depending on the drying method, we next examined two drying methods, natural evaporation drying and vacuum drying, which have been commonly used for microneedle fabrication. Microneedles were coated with F4 (smallpox + PVA + trehalose) three times, dried using different methods, and subjected to virus quantification. As shown in Figure 3, the viral titer of the vacuum-dried microneedles was significantly higher than that of the naturally dried microneedles, suggesting that fast drying would be beneficial in maintaining the stability of the vaccinia virus. Additionally, we anticipate that the vacuum-dried samples would show better long-term stability than the naturally dried samples due to lower moisture content, which is a key contributor to vaccine stability [36]. When dried naturally, the moisture contained in the coating layer evaporates slowly due to the lack of driving force and solidification of the coating surface. In contrast, vacuum drying accelerates moisture evaporation and lowers the moisture content in the coating layer compared to natural drying. Low moisture content in the coating layer decreases the mobility of the vaccine, contributing to the stabilization of the vaccine in the dried state. Moreover, a previous study suggested that slow drying could cause denaturation of virus, thereby lowering the potency of the smallpox vaccine [37].

### 3.2. Influence of Excipients on Vaccine Storage Stability

**Figure 4:**
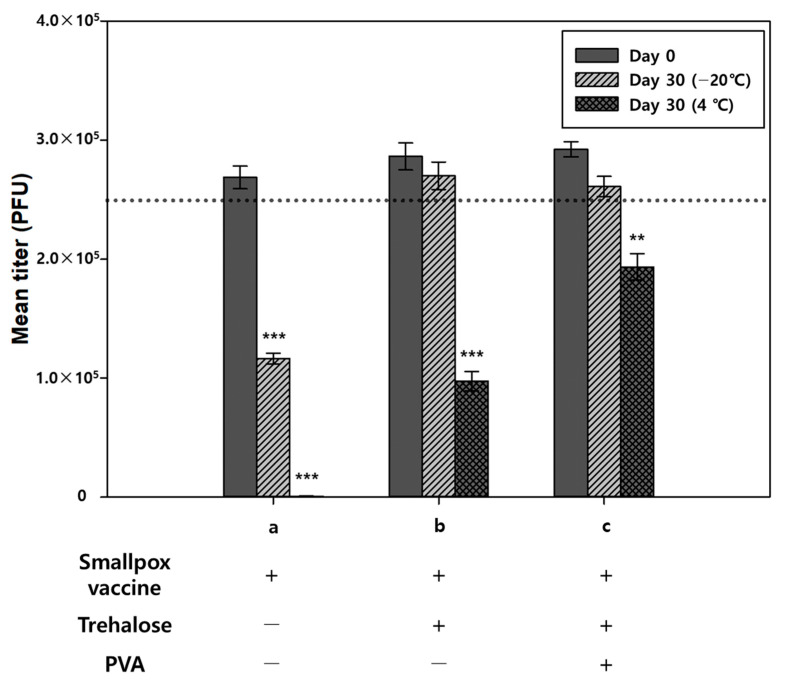
One-month storage stability of vaccine-coated microneedles stored at 4 °C and −20 °C. (**a**) Smallpox vaccine only (F1), (**b**) smallpox vaccine + trehalose (F3), and (**c**) smallpox vaccine + polyvinyl alcohol (PVA) + trehalose (F4). Dotted line indicates the targeted virus coating amount. Statistical significance compared to the Day 0 sample was determined by a *t*-test (** *p* < 0.01, *** *p* < 0.001).

We further examined the stability of the vaccine during storage when formulated with the excipients. Since our preliminary stability study using a PLA chip that mimics the surface of microneedle showed that trehalose is beneficial in maintaining the stability of the smallpox vaccine when stored at 4 °C for 30 days (Figure S1), we prepared vaccine-coated microneedles with the formulations containing trehalose, stored them at 4 °C and −20 °C for 30 days, and examined the change in viral titers. When stored at 4 °C for 30 days, there was no detectable titer in the samples without excipients (Figure 4, group a), whereas the samples containing excipients showed measurable titers (Figure 4, groups b and c). In particular, the formulation containing the combination of PVA and trehalose had less loss of titer than the formulation of trehalose alone (Figure 4, group c). We anticipate that the addition of polymers, such as PVA, would delay the crystallization of trehalose during storage, maximizing the stabilizing effect of trehalose [38].

Compared to the viral titer right after solidification, storage at −20 °C for 30 days caused additional 56.7% reduction in titer (Figure 4, group a). This reduction may be due to freeze–thaw stress and ice crystal formation during reconstitution and storage [39,40,41]. When trehalose was included in the vaccine formulation, the viral titer was well maintained (Figure 4, group b and c), suggesting that trehalose acts as a good cryoprotectant for the vaccina virus. Notably, the addition of PVA to the formulation appeared not to be effective in retaining the virus titers under frozen storage conditions compared with the trehalose-only formulation. However, the PVA–trehalose formulation showed improved storage stability at 4 °C. We speculate that PVA might not be able to protect the virus from freeze–thaw stress; however, it might be capable of reducing stress during reconstitution, resulting in similar viral titer retention in both PVA-only and PVA–trehalose formulations stored at −20 °C and better viral titer retention in the PVA-only formulation stored at 4 °C (Figure S1).

### 3.3. Long-Term Stability Test

**Figure 5:**
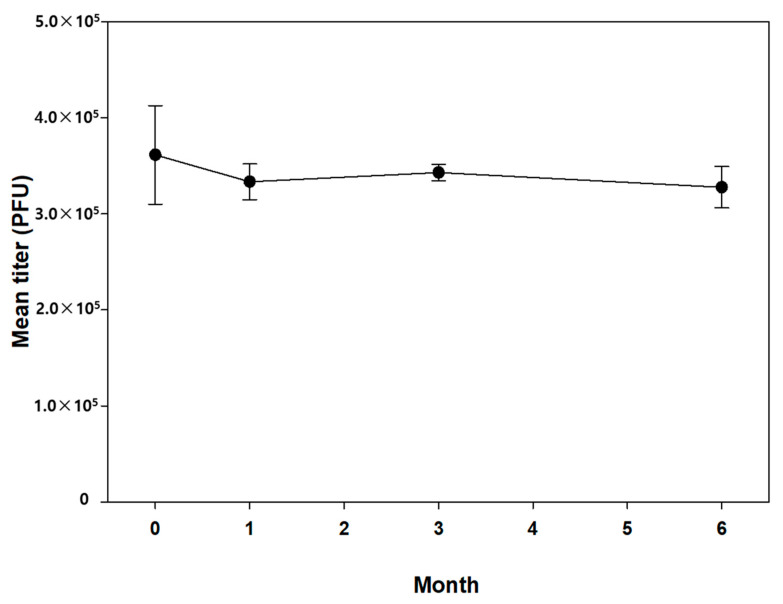
Long-term stability of smallpox-vaccine-coated microneedle stored at −20 °C. Statistical significance compared to the Day 0 sample was determined by a *t*-test. There is no statistically significant difference (*p* = 0.860).

Since the stability of vaccine-coated microneedles was well maintained at −20 °C for a month, we further examined the change in virus titer for 6 months under the same storage conditions. For this study, the microneedles were coated with 3.3 × 10^5^ PFU of the vaccinia virus, which is higher than the human dose (2.5 × 10^5^ PFU), considering the typical delivery efficiency of microneedles (approximately 80%) [42,43]. PVA (5% *w*/*v*) and trehalose (15% *w*/*v*) were used as excipients, and the vaccine-coated microneedles were dried under vacuum to minimize virus titer reduction. As shown in Figure 5, the virus titer appeared to be slightly reduced during the first month of storage but there was no statistically significant difference. In addition, the virus titer was maintained over 6 months.

Although the current formulation was able to maintain the stability of the smallpox vaccine coated on microneedles for up to 6 months at −20 °C, it will be desirable to develop a highly stable formulation that can be stored at higher temperatures. In this study, we selected 5% PVA and 15% trehalose as excipients on the basis of our previous results and reports from the literature because of the limited amount of vaccine available for the experiments. We anticipate that it would be possible to develop a thermostable smallpox-vaccine-coated microneedle by exploring additional excipients and process parameters. For example, a lyophilized smallpox vaccine was reported to be stable for 12 months and 4 months when stored at 22 °C and 37 °C, respectively [37].

### 3.4. Immunogenicity of Smallpox-Vaccine-Coated Microneedles in Mice

**Figure 6:**
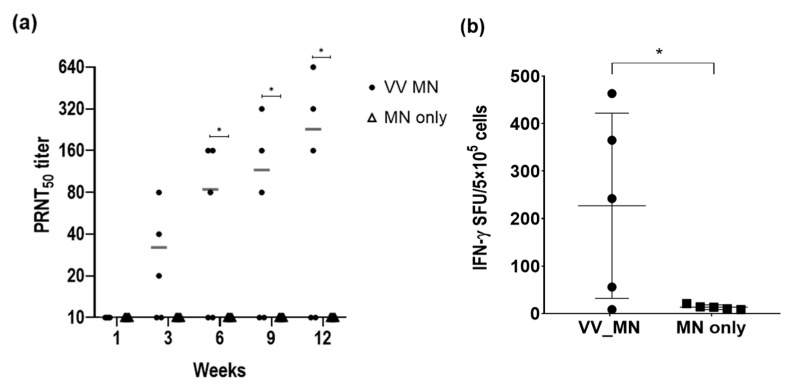
Humoral and cell-mediated immune responses after vaccination using microneedles. (**a**) PRNT50 titer of neutralizing antibody against the vaccinia virus, and (**b**) interferon (IFN)-γ-secreting cells were evaluated using the enzyme-linked immunospot (ELISPOT) assay for analyzing T-cell-mediated immune response. Horizontal bars in each graph indicate geometric mean titer. Statistical significance compared to MN only group was determined by a *t*-test (* *p* < 0.05). PRNT, plaque reduction neutralisation test; SFU, spot forming unit.

To evaluate the efficacy of smallpox-vaccine-coated microneedles, mice (*n* = 5) were immunized with microneedles coated with 3.3 × 10^5^ PFU vaccinia virus. Blank microneedles were used as a control. Serum samples were collected at 1, 3, 6, 9, and 12 weeks after immunization, and neutralizing antibodies against the vaccinia virus were analyzed by PRNT. As shown in Figure 6a, neutralizing antibodies were elicited beginning 3 weeks after immunization and were maintained for 12 weeks. Furthermore, we examined cell-mediated immune responses induced by microneedle vaccination (Figure 6b); 12 weeks after priming, splenocytes collected from the mice were stimulated with UV-inactivated vaccinia virus for 2 days, and IFN-γ-secreting cells were measured by the ELISPOT assay to analyze T cell-mediated immune responses. We observed a significant increase in the IFN-γ-secreting cells after vaccination with the microneedles compared to that in the blank microneedle injection group. These results indicate that smallpox-vaccine-coated microneedles could elicit both humoral and cellular immune responses with a single immunization at least for 12 weeks in mice.

In some of the vaccinated mice, neutralizing antibodies were not induced and minimal cell-mediated responses were observed. We speculate that variation in microneedle insertion depth by the thumb pressure application technique might have attributed to the difference in immune responses. A previous report showed that it is difficult to induce immune responses if vaccina virus is administered too deeply or too superficially [44]. We anticipate that this problem could be overcome by ensuring consistent needle insertion depth, which would be feasible by adopting an applicator and adjusting the length of microneedles.

Additional data are needed to evaluate whether microneedle vaccination is more effective than other vaccination methods such as IM and SC injection. Although most vaccines are administered through IM or SC route, IM delivery of vaccinia virus was ineffective in protecting against smallpox [45]. A previous study showed that skin scarification was the most effective approach for eliciting memory T cell responses and generating neutralizing antibodies against smallpox virus compared with IM, intradermal, intraperitoneal, and SC injections [46]. We anticipate that smallpox vaccination using microneedles would be a more effective and appropriate method than conventional needle injection methods because appropriately designed microneedles could achieve both skin scarification and intradermal injection.

## 4. Conclusions

In this study, we examined the feasibility of smallpox vaccination using microneedles. Stability tests suggest that fabrication processes, such as thawing, mixing, coating, and drying, would adversely affect the virus viability, reducing the potency of live virus vaccines. Our data indicate that fast drying processes, such as vacuum drying, would be helpful in retaining the viability of the vaccina virus. The use of stabilizers did not effectively protect the virus from degradation during solidification; however, it significantly helped maintain the stability of the virus during storage. Therefore, exploring various combinations of excipients that can minimize the stress induced during drying would enable us to improve the vaccine stability during solidification. In addition, our stability data demonstrated that the combination of PVA and trehalose is suitable for coating, and it can maintain the virus titers up to 6 months when stored at −20 °C. Furthermore, the developed smallpox vaccine-coated microneedle induced both humoral and cell-mediated immune responses in the mice, suggesting the potential of smallpox vaccination by microneedles. Although microneedles coated with the developed vaccine formulation would provide advantages over lyophilized smallpox vaccines, including reduced storage space, single-dose form for minimizing vaccine wastage and contamination, and less pain during administration, it is highly desirable to develop a thermostable smallpox microneedle that can be stored at elevated temperatures over extended periods of time. We expect that, compared to conventional vaccination by bifurcated needles, microneedle-based smallpox vaccination would not only provide less complicated vaccine administration with higher dosing accuracy but also reduce economic burden associated with vaccine storage and distribution. In conclusion, microneedles could serve as a potential alternative method for smallpox vaccination and stockpiling and could allow us to respond rapidly to emergencies such as bioterrorism.

## Figures and Tables

**Table 1:** Composition of coating solutions.

Formulation	Smallpox Vaccine	PVA	Trehalose	PBS
F1	2.5 × 10^7^ PFU	−	−	300 μL
F2	2.5 × 10^7^ PFU	5% (*w*/*v*)	−	300 μL
F3	2.5 × 10^7^ PFU	−	15% (*w*/*v*)	300 μL
F4	2.5 × 10^7^ PFU	5% (*w*/*v*)	15% (*w*/*v*)	300 μL

## Data Availability

The data that support the findings of this study are available from the corresponding authors upon reasonable request.

## Supplementary Materials

The following are available online at https://www.mdpi.com/1999-4923/13/2/209/s1, Figure S1: One-month storage stability of solidified vaccine stored at 4 °C.
