# Supplementary material for: Live Vaccinia Virus-Coated Microneedle Array Patches for Smallpox Vaccination and Stockpiling
Source: Pharmaceutics. 2021 Feb 3;13(2):209. doi: 10.3390/pharmaceutics13020209 (PMC7913550; doi:10.3390/pharmaceutics13020209)
Supplement: Supplementary file 1 [file pharmaceutics-13-00209-s001.pdf]

pharmaceutics

MDPI

Supplementary Materials: Live Vaccinia Virus-Coated Microneedle Array Patches for Smallpox Vaccination and Stockpiling

In-Jeong Choi, Hye-Ran Cha, Su Jin Hwang, Seung-Ki Baek, Jae Myun Lee and Seong-O Choi

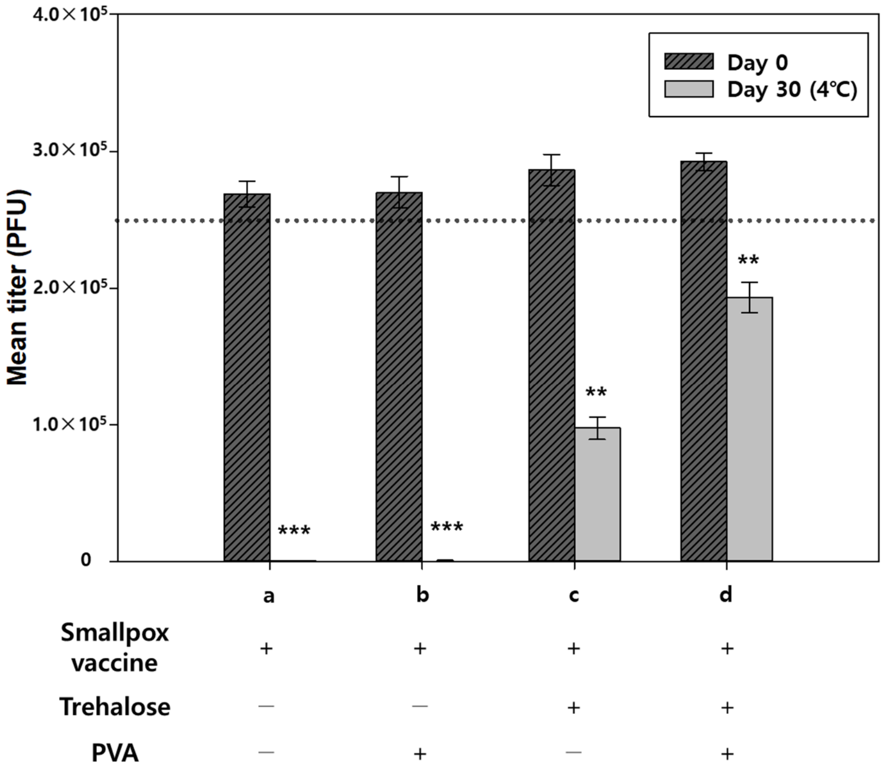

Figure S1. One-month storage stability of solidified vaccine stored at 4 °C. Phosphate-buffered saline (200 μL) was added to the formulations (described in Table 1) for preparing samples at 5 × 10^7 PFU/mL, and 10 μL of each sample was dried under ambient conditions on polylactic acid (PLA) chips that simulate the surface of PLA microneedles. Because approximately 40% of the virus titer was lost during solidification (Figure 2), we anticipated that the virus titer of each sample would range between 2.5 × 10^7 and 3.0 × 10^7 PFU/mL after reconstitution of the solidified samples. The prepared samples were stored at 4 °C for 30 days, and the virus titer was examined. (**a**) smallpox vaccine only, (**b**) smallpox vaccine + polyvinyl alcohol (PVA), (**c**) smallpox vaccine + trehalose, and (**d**) smallpox vaccine + PVA + trehalose. Dotted line indicates virus titer in the stock solution. Statistical significance compared to the Day 0 sample was determined by a t-test (**$p < 0.01$, ***$p < 0.001$).

Citation: Choi, I.-J.; Cha, H.-R.; Hwang, S.J.; Baek, S.-K.; Lee, J.M.; Choi, S.-O. Live vaccinia virus-coated microneedle array patches for smallpox vaccination and stockpiling. *Pharmaceutics* **2021**, *13*, 209. https://doi.org/10.3390/pharmaceutics13020209

Received: 25 November 2020
Accepted: 30 January 2021
Published: 3 February 2021

Publisher's Note: MDPI stays neutral with regard to jurisdictional claims in published maps and institu-tional affiliations.

Copyright: © 2021 by the authors. Submitted for possible open access publication under the terms and conditions of the Creative Commons Attribution (CC BY) license (http://creativecommons.org/licenses/by/4.0/).
